# MYC and DNMT3A‐mediated DNA methylation represses microRNA‐200b in triple negative breast cancer

**DOI:** 10.1111/jcmm.13916

**Published:** 2018-10-16

**Authors:** Yamei Pang, Jian Liu, Xiang Li, Guodong Xiao, Huangzhen Wang, Ganghua Yang, Yanbo Li, Shou‐Ching Tang, Sida Qin, Ning Du, Henggang Zhang, Dapeng Liu, Xin Sun, Hong Ren

**Affiliations:** ^1^ Department of Respiratory and Critical Care Medicine The First Affiliated Hospital of Xi'an Jiaotong University Xi'an Shaanxi Province China; ^2^ Department of Thoracic Surgery and Oncology The Second Department of Thoracic Surgery Cancer Center The First Affiliated Hospital of Xi'an Jiaotong University Xi'an Shaanxi Province China; ^3^ Department of Surgical Oncology Baoji Central Hospital Baoji Shaanxi Province China; ^4^ Department of Geriatric Surgery The First Affiliated Hospital of Xi'an Jiaotong University Xi'an Shaanxi Province China; ^5^ Department of Neurology The First Affiliated Hospital of Xi'an Jiaotong University Xi'an Shaanxi Province China; ^6^ Breast Cancer Program and Interdisciplinary Translational Research Team Georgia Regents University Cancer Center Augusta Georgia; ^7^ Tianjin Medical University Cancer Institute and Hospital Tianjin China; ^8^ Department of Thoracic Surgery and Oncology People's Hospital of Hanzhong City Hanzhong Shaanxi Province China

**Keywords:** DNA methylation, DNMT3A, miR‐200b, MYC, TNBC

## Abstract

Triple‐negative breast cancer (TNBC) is the most aggressive breast cancer subtype with a poor prognosis. The microRNA‐200 (miR‐200) family has been associated with breast cancer metastasis. However, the epigenetic mechanisms underlying miR‐200b repression in TNBC are not fully elucidated. In this study, we found that MYC proto‐oncogene, bHLH transcription factor (MYC) and DNA methyltransferase 3A (DNMT3A) were highly expressed in TNBC tissues compared with other breast cancer subtypes, while miR‐200b expression was inhibited significantly. We demonstrated that MYC physically interacted with DNMT3A in MDA‐MB‐231 cells. Furthermore, we demonstrated that MYC recruited DNMT3A to the miR‐200b promoter, resulting in proximal CpG island hypermethylation and subsequent miR‐200b repression. MiR‐200b directly inhibited DNMT3A expression and formed a feedback loop in TNBC cells. MiR‐200b overexpression synergistically repressed target genes including zinc‐finger E‐box‐binding homeobox factor 1, Sex determining region Y‐box 2 (SOX2), and CD133, and inhibited the migration, invasion and mammosphere formation of TNBC cells. Our findings reveal that MYC can collaborate with DNMT3A on inducing promoter methylation and miR‐200b silencing, and thereby promotes the epithelial to mesenchymal transition and mammosphere formation of TNBC cells.

## 1 INTRODUCTION

Breast cancer is a kind of heterogeneous disease, being classified into distinct subtypes based on the molecular markers variants. Triple‐negative breast cancer (TNBC), characterized by the absence of the oestrogen receptor (ER), the progesterone receptor (PR), and the human epidermal growth factor receptor 2 (HER2) expression, was known as the most aggressive breast cancer subtype.^1^ TNBC patients have a higher risk of distant metastasis and a poorer overall survival than other breast cancer patients, partly due to lacking effective targeted therapies.^2^ Despite the great effort made on researching TNBC, the molecular mechanisms underlying TNBC aggressive behaviour are still obstacles to TNBC treatment.

MicroRNAs (miRNAs) are small non‐coding RNAs that function through binding to the 3′‐untranslated region (3′‐UTR) of target mRNAs, resulting in mRNA degradation or translation inhibition.^3^, ^4^, ^5^ Increasing evidence has revealed that deregulated miRNAs are involved in human carcinogenesis and cancer stem‐like cells by interacting with the downstream genes.^4^, ^6^, ^7^, ^8^ For example, the repression of miR‐200 family plays a critical role in the process of cancer metastasis.^9^, ^10^ MiR‐200 family consists of five members transcribed from two clusters. One cluster located on chromosome 1 encodes miR‐200b/200a/429, and the other on chromosome 12 encodes miR‐200c/141.^11^ MiR‐200 family members inhibit epithelial‐to‐mesenchymal transition (EMT) by directly targeting zinc‐finger E‐box‐binding homeobox factor 1 (ZEB1) and 2 (ZEB2).^12^, ^13^, ^14^

Recent reports have demonstrated that miR‐200 expression is inhibited by several transcription factors.^11^ ZEB1 and ZEB2 repress the miR‐200 promoter activity by binding to the E‐box elements, suggesting a feedback loop with miR‐200.^15^, ^16^, ^17^ Apart from transcription factors aforementioned, epigenetic mechanisms, including DNA methylation as well as histone methylation and acetylation are all implicated in miR‐200 silencing.^18^, ^19^, ^20^ For example, DNA methyltransferase 1 (DNMT1) and EZH2 mediated DNA methylation and H3K27 trimethylation silence miR‐200b/200a/429 gene and contribute to the progression of gastric cancer and glioblastoma.^21^ However, few studies have focused on the association of miR‐200b repression with DNMT3A‐mediated DNA methylation, which is crucial for digging out the mechanisms of DNA methylation in regulating miRNAs.^22^

Previous studies have demonstrated that the activation of MYC proto‐oncogene, bHLH transcription factor (MYC) gene contributes to cancer genesis.^23^, ^24^ However, the roles of MYC in epigenetically regulating miRNAs are largely unclear. In this study, we aimed to reveal the mechanism of MYC and DNMT3A inducing miR‐200b repression, which was involved in EMT and mammosphere formation of TNBC cells.

## 2 MATERIALS AND METHODS

### 2.1 Cell culture and treatment

Human breast cancer cell lines MCF‐7, ZR75‐1, T47D, SKBR‐3, MDA‐MB‐231 (MM‐231), BT549, Hs578T and normal breast cell line MCF‐10A were obtained from American Type Culture Collection (ATCC, Manassas, VA, USA) and kept in the Center for Translational Medicine of the First Affiliated Hospital of Xi'an Jiaotong University. Briefly, breast cancer cells were maintained at 37°C, 5% CO_2_ in DMEM medium (Invitrogen, Carlsbad, CA, USA) supplemented with 10% foetal bovine serum (Hyclone, Salt Lake City, UT, USA) and 1% penicillin/streptomycin (Cellgro, Herndon, VA, USA). MCF‐10A cell line was cultured in DMEM/F12(1:1) (Hyclone) supplemented with 5% FBS, 10 μg/mL insulin, 20 ng/mL EGF, 100 ng/mL cholera toxin and 0.5 mg/mL hydrocortisone at 37°C, 5% CO_2_. The MDA‐MB‐231 and BT549 cells were treated with 5‐Aza‐2′‐deoxycytidine (Decitabine, DAC; Sigma‐Aldrich Corporation, St. Louis, MO, USA) at a final concentration of 5 μM. The medium containing DAC was refreshed every 24 hours. Control group cells were treated with the dimethylsulfoxide at a concentration of 0.001%.

### 2.2 Cell transfection

MiR‐200b mimics, miR‐200b inhibitors, DNMT3A siRNA, MYC siRNA and their corresponding negative controls were chemically synthesized by RiboBio (Guangzhou, China). MDA‐MB‐231 or BT549 cells were seeded into six‐well plates at 1 × 10^6^ cells/well for overnight and transfected with miR‐200b mimics (50 nM), miR‐200b inhibitors, siRNA (100 nM) or the corresponding negative control. MiRNA‐200b overexpression lentiviral vectors and scramble vectors (pGLVU6/RFP) were constructed by GenePharma (Shanghai, China). All transfections were conducted using Lipofectamine 2000 (Invitrogen) following the manufacturer's protocol.

### 2.3 Tissue specimens

All tissue samples (ER+, n = 20; ER‐PR‐HER2+, n = 13; TNBC, n = 31) used in this study were obtained from patients of the First Affiliated Hospital of Xi'an Jiaotong University (Xi'an, China) between June 2015 and February 2017. This study was approved by the Ethics Committee of Xi'an Jiaotong University First Affiliated Hospital (XJTU1AF2016LSK‐05) and a written informed consent document was signed by each participant. The specimens were resected and frozen in liquid nitrogen immediately after surgery. None of the patients had received any chemotherapy or radiation therapy prior to the study. Clinicopathologic characteristics of the patients were shown in Table S1. The classification of the tumours as TNBC was determined according to 2009 St. Gallen's Consensus guidelines for ER and PR markers and ASCO/CAP 2013 guidelines for HER2 testing.

### 2.4 Quantitative real‐time PCR

The total RNA was isolated from cells using TRIzol^®^ Reagent (Invitrogen) according to the manufacture's protocol. Approximately 1 μg total RNA was reverse‐transcribed using PrimeScriptTM RT reagent Kit (Takara Biotechnology, Dalian, China). The real‐time PCR was conducted with SYBR® Premix Ex Taq™ II (Tli RNaseH Plus) (2×) (Takara Biotechnology) on a CFX96TM Real‐Time PCR Detection System (Bio‐Rad, Hercules, CA, USA) according to the manufacturer's instructions. β‐actin and U6 were used as internal control for MYC, DNMT3A and miR‐200B respectively. The relative expression levels of the target genes were analysed using the comparative threshold cycle (2^−ΔΔCt^) method as previously described.^25^ The primers for real‐time PCR are provided in Table S2.

### 2.5 Immunofluorescence and immunohistochemistry staining

The cells were fixed in 4% formaldehyde in PBS for 15 minutes, permeabilized with 0.2% Triton X‐100 for 20 minutes and blocked with bovine serum albumin at 37°C for 30 minutes. Fixed cells were incubated with the antibodies against DNMT3A (1:200, IMG268; Imgenex, San Diego, CA, USA) at 4°C overnight, followed by Alexa Fluor 594 goat anti‐rabbit IgG (H+L) Secondary Antibody (1:1000, A‐11012; Life Technologies, Gaithersburg, MD, USA) and Alexa Fluor 488 Goat antimouse IgG (H+L) Secondary Antibody (1:1000, A‐11029; Life Technologies) for 1 hour at room temperature. The nuclei were counterstained with 4,6‐diamidino‐2‐phenylindole (DAPI, 1:10 000; 4084; Cell Signaling Technology, Beverly, MA, USA). The fluorescence images were captured with a confocal laser microscope.

Immunohistochemical analysis for MYC and DNMT3A expression of TNBC tissues and normal adjacent breast tissues was performed as we previously described.^7^, ^25^ Each section was examined under a light microscope to ensure >70% tumour content. Primary antibodies and dilutions for immunohistochemistry (IHC) are as follows: anti‐MYC antibody (1:100, N262; Santa Cruz Biotechnology, Santa Cruz, CA, USA), anti‐DNMT3A antibody (1:200, IMG268; Imgenex). Five random fields without overlaps of each slide were selected to evaluate staining intensity. The negative control was performed as above without using primary antibody. Previously tested TNBC samples (DNMT3A or MYC‐positive) were used as positive control. A modified H‐score was used for the quantification of IHC staining. Both the staining intensity and the proportion of positive cell were taken into consideration. It was defined as follows: 0, no staining; 1, weakly staining, light brown staining in nucleus; 2, moderately staining, brown staining in nucleus; 3, strongly staining, dark brown staining of nucleus. H‐score=no staining cells%×0 + weakly staining cells%×1 + moderately staining cells%×2 + strongly staining cells%×3.

### 2.6 Western blot

Cells were lysed in RIPA buffer (Thermo Scientific, Waltham, MA, USA) with Protease Inhibitor Cocktail Tablets (Roche, Mannheim, Germany) for 15 minutes on ice. The total protein concentrations were measured using Protein BCA Assay Kit (Bio‐Rad). Samples were denatured with 5× loading buffer at 100°C for 5 minutes. Equal amounts of protein (25 μg) were loaded on a 10% SDS‐PAGE gel. The protein in the lysates were resolved by electrophoresis and transferred onto NC membranes (Bio‐Rad). The membrane was blocked for 2 hours at room temperature in 5% nonfat milk, and subsequently incubated with primary antibody as follows: DNMT3A(1:1000, IMG268; Imgenex), MYC (1:1000, N262; Santa Cruz Biotechnology), Sex determining region Y‐box 2 (SOX2, 1:1000, ab92494; Abcam, Cambridge, MA, USA), CD133(1:200, 130105226; Miltenyi Biotec, Bergisch Gladbach, Germany), ZEB1(1:1000, ab203829; Abcam) and E‐cadherin (1:1000, 3195; Cell Signaling Technology). Then, the membranes were incubated with HRP‐conjugated secondary antibody (1:5000; Santa Cruz Biotechnology). An anti‐β‐actin antibody (1:5000, A5441; Sigma‐Aldrich Corporation) was used as internal control.

### 2.7 Methylation‐specific PCR and Bisulfite sequencing

Genomic DNA was extracted from cells using TIANamp Genomic DNA Kit (TIANGEN, Beijing, China) according to manual instructions. The conversion of DNA by sodium bisulfite was performed with EZ DNA Methylation‐Gold™ Kit (Zymo Research, Irvine, CA, USA) following the manufacturer's instructions. The sodium bisulfite‐converted DNA was amplified with TaKaRa Taq™Hot Start Version (Takara Biotechnology). The primers for methylation‐specific PCR (MSP) were as follows: methylated forward primer, 5′‐GAGCGGAGATTGGTTAGC‐3′; reverse primer, 5′‐TCGAAAACGACGAAACAATAA‐3′; unmethylated forward primer, 5′‐TAGGAGTGGAGATTGGTTAGT‐3′; reverse primer, 5′‐AAATTTCAAAAACAACAAAACAAT‐3′. The primers for sequencing region of miR‐200b promoter were as follows: forward primer, 5′‐TGGGAGTTTAGGGGATATATTTG‐3′; reverse primer, 5′‐TCTACCTCAACCAAAATCAAACC‐3′. At least six clones were subjected to sequencing analyses (Invitrogen, Shanghai, China).

### 2.8 Dual‐Luciferase Reporter

The 3′‐UTR of DNMT3A mRNA containing the wild‐type or mutant miR‐200b‐binding site were chemically synthesized by Invitrogen (China), and inserted into the pGL3‐control Vector (Promega, Madison, WI, USA). MDA‐MB‐231 cells were seeded in 24‐well plates and cotransfected with 0.2 μg firefly luciferase report vector, 0.04 μg control vector containing Renilla luciferase, pRL‐TK (Promega), and 50 nM miR‐200b mimics or negative control mimics using Lipofectamine 2000 (Invitrogen) according to the manufacturer's instructions. 48 hours after transfection, the firefly and *Renilla* luciferase were detected with Dual‐Luciferase Reporter Assay System (Promega). The relative luciferase activity was normalized by renilla luciferase activity. Each experiment was performed in triplicate.

### 2.9 Co‐immunoprecipitation assays

Co‐immunoprecipitation was conducted as preciously described.^26^ Briefly, cells were rinsed with ice‐cold PBS and lysed in RIPA buffer. The cells lysates were pre‐cleared by adding 100 μL of Protein A/G agarose beads, and then subjected to overnight incubation with specific antibodies anti‐MYC antibody (N262; Santa Cruz Biotechnology) or anti‐DNMT3A antibody (IMG268; Imgenex). Immunocomplexes were precipitated by incubating with Protein A/G agarose beads, followed by washing with ice‐cold lysis buffer. Subsequently, the agarose beads with immune complexes were boiled in 2× SDS sample buffer. Then, the immune complexes were analysed by western blotting.

### 2.10 Chromatin immunoprecipitation assay

Chromatin immunoprecipitation (ChIP) was performed as previously described with minor modification.^18^, ^26^ Briefly, cells were cross‐linked with 1% formaldehyde at 37°C for 10 minutes. After removing the medium, cells were resuspended in 100 μL lysis buffer per 1 × 10^6^ cells and lysed on ice for 15 minutes. Then, the samples were sonicated to shear chromatin to fragments ranging from 200 to 1000 base pairs. Subsequently, the chromatin fragments were used for immunoprecipitation with antibodies as follows: anti‐MYC antibody (N262; Santa Cruz Biotechnology) and anti‐DNMT3A antibody (IMG268; Imgenex). The primers used for PCR analysis for the proximal promoter region of the miR‐200b were as follows: forward primer, 5′‐CACCGCCTCCCATTGTC‐3′; reverse primer, 5′‐CACAGGAAGTCAGTTCAGACC‐3′.

### 2.11 Sphere‐formation assays

Mammospheres were cultured as we previously described.^7^, ^18^ Cells were grown in serum‐free mammosphere medium (DMEM/F12) in ultra‐low attachment dishes (Corning Incorporated, Lowell, MA, USA), supplemented with 1:50 B27 (Invitrogen, USA), 20 ng/mL recombinant human basic FGF (Invitrogen, USA), 5 μg/mL insulin, 0.5 mg/mL hydrocortisone and 20 ng/mL epidermal growth factor (Invitrogen, USA). After 10 days for culturing, mammospheres >50 μm were counted. The mammosphere forming efficiency was calculated as the ratio of obtained spheres verses number of plated cells (mammospheres/1000 cells). All mammosphere experiments were performed in triplicate independently.

### 2.12 Migration and invasion assays

The cells were planted in six‐well plates and transfected as indicated. The scratch wound was created using a 200 μL pipette tip on the confluent cell monolayer. The scratched cellular monolayer was washed with fresh medium to remove the floating cells. After culturing for 24 hours, the spread of wound healing was observed and photographed under a microscope. The ImageJ software (National Institutes of Health, Baltimore, MD, USA) was used to analyse the migration distance.

Transwell chamber (8 μm pore size; Corning Inc.) coated with extracellular matrix gel (BD Biosciences, San Jose, CA, USA) was used to determine the invasion capacity in vitro. After transfection, MDA‐MB‐231 cells were seeded into the upper chamber with serum‐free medium. After incubation for 24 hours, the non‐invading cells on the upper surface of the membrane were removed with a cotton swabs, and the invading cells on the lower surface were fixed with methanol and stained with crystal violet. The number of invading cells was counted in five random fields for each membrane at ×400 magnification.

### 2.13 Statistical analysis

All statistical analyses were performed with SPSS software (version 18.0; SPSS Inc., Chicago, IL, USA). All data were represented as mean ± SD from at least three independent experiments. The statistical significance in mean values was determined with Student's *t*‐test (two‐tailed). For skewed distribution data, statistical significance was determined with nonparametric test. *P* < 0.05 indicated a statistically significant difference.

## 3 RESULTS

### 3.1 High MYC, DNMT3A levels and low miR‐200b levels in TNBC

**Figure 1:**
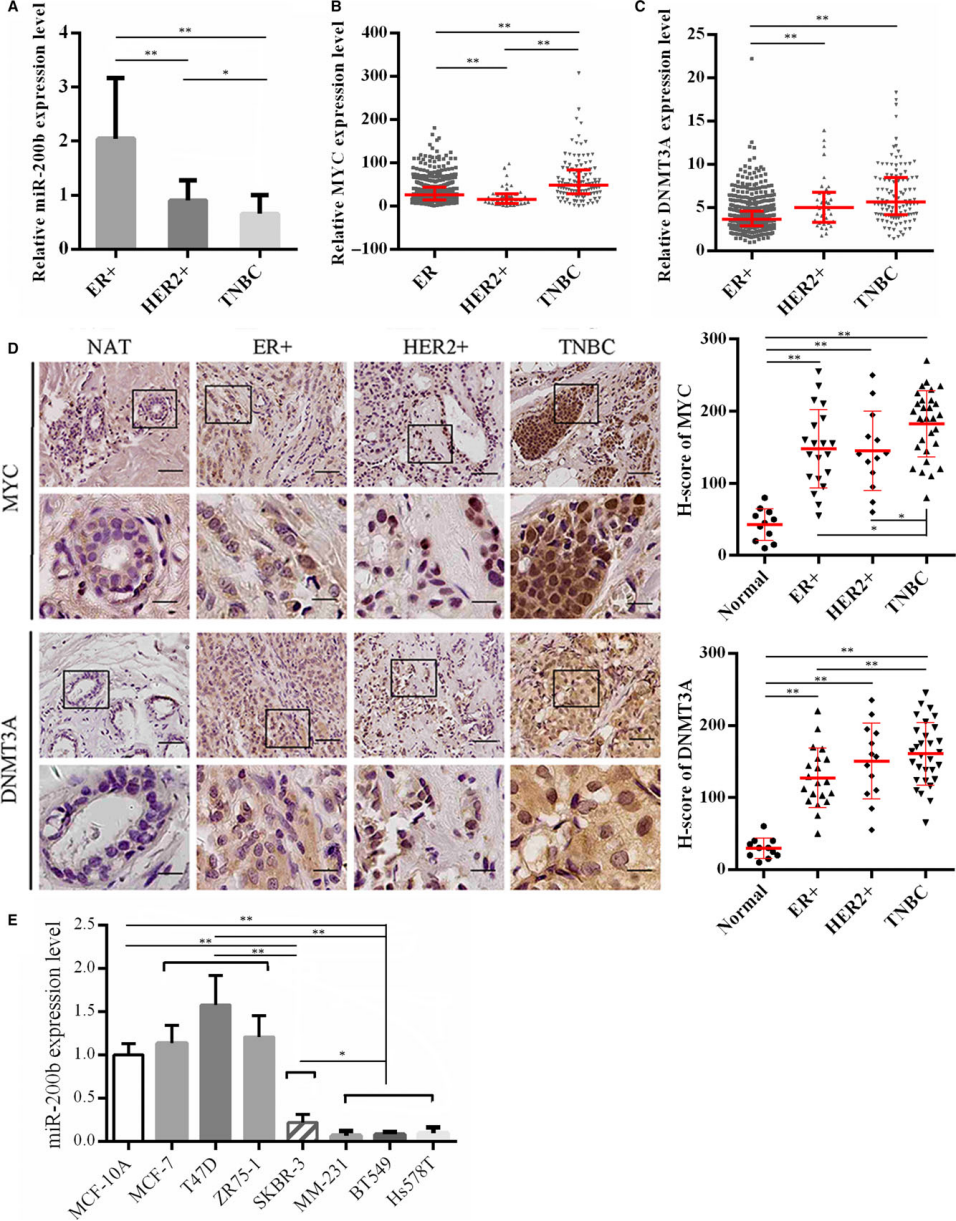
The expression of MYC, DNMT3A and miR‐200b in breast cancer. (A) MiR‐200b levels in ER+ (n = 20), HER2+ breast cancer (n = 13) and TNBC tissues (n = 31) were analysed by qRT‐PCR. U6 was used as internal control. The results are presented as mean ± SD. (B and C) MYC and DNMT3A expression data in TCGA RNA‐Sequencing in ER+ (n = 803), HER2+ breast cancer (n = 37) and TNBC tissues (n = 115). The results are presented as median with interquartile. (D) The expression of MYC and DNMT3A protein were detected by immunohistochemistry in ER+ (n = 20), HER2+ breast cancer (n = 13), TNBC tissues (n = 31) and normal adjacent barest tissues (n = 11). Representative Immunohistochemistry images are shown. The H‐score are presented as mean ± SD. Scale bar: upper 50 μm, lower 15 μm. (E) miR‐200b levels in breast cancer cell lines and normal breast cell line were detected by qRT‐PCR. NAT, normal adjacent breast tissue; HER2+, ER‐PR‐HER2+ breast cancer; TNBC, triple negative breast cancer. The experiments were repeated in triplicate. *P < 0.05; **P < 0.01

We began the study with evaluating MYC, DNMT3A and miR‐200b levels in breast cancer tissues. We found that miR‐200b expression in TNBC was reduced compared with in ER+ and ER‐PR‐HER2+ (simplified as HER2+) breast cancer (Figure 1A). The RNA‐sequencing data of TCGA showed DNMT3A and MYC were highly expressed in TNBC tissues compared with in other breast cancer subtypes (Figure 1B and C). The IHC analysis showed that MYC and DNMT3A were up‐regulated in TNBC tissues than other breast cancer subtypes (H‐score of MYC, ER+, 148.0 ± 12.2; HER2+, 145.2 ± 15.3; TNBC, 182.5 ± 8.3; H‐score of DNMT3A, ER+, 127.3 ± 9.3; HER2+, 150.5 ± 14.6; TNBC, 160.6 ± 7.9) (Figure 1D and S1A).

We then detected the endogenous levels of miR‐200b in multiple breast cancer cell lines, and identified lower expression levels of miR‐200b in TNBC cells (MDA‐MB‐231, BT549 and Hs578T) compared with breast epithelial cell line of MCF‐10A and other breast cancer cell lines (Figure 1E). Moreover, miR‐200b methylation levels in TNBC cells were higher than other breast cancer cells as previously described.^20^, ^27^, ^28^ As a result, the following experiments were mainly performed in two TNBC cell lines, MDA‐MB‐231 and BT549.

However, MYC and DNMT3A levels in breast cancer cell lines did not show a significant subtype‐specific signature (Figure S1B and C). In addition, there was no significant difference in miR‐200b promoter methylation levels among the breast cancer subtypes according to TCGA methylation database (Figure S1D).

### 3.2 DNMT3A knockdown induces miR‐200b expression via promoter demethylation

**Figure 2:**
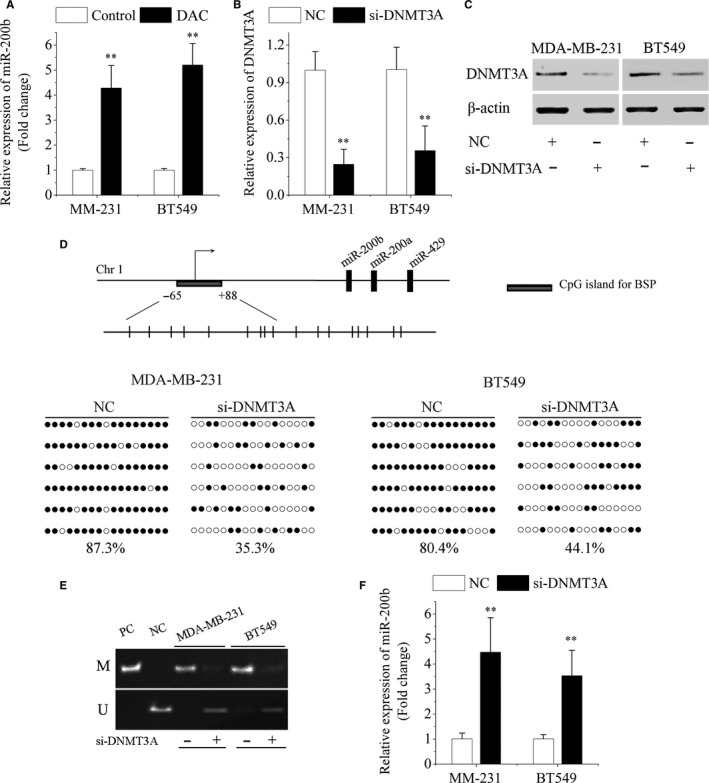
DNMT3A knockdown increases miR‐200b expression via promoter demethylation. (A) Treatment with DAC increased miR‐200b expression levels in MDA‐MB‐231 and BT549 cells. qRT‐PCR (B) and Western blot (C) assay of DNMT3A expression in MDA‐MB‐231 and BT549 cells after transfection with DNMT3A siRNA (si‐DNMT3A) or negative control (NC). (D) A schematic diagram of the CpG sites in the miR‐200b promoter for bisulfite sequencing was shown. The arrow indicated the transcription start site. The methylation levels of the miR‐200b promoter were analysed by bisulfite sequencing. The unmethylated or methylated CpG sites were indicated by white or black circles respectively. Six single clones per group were sequenced. (E) The promoter methylation status of miR‐200b was analysed following transfection with si‐DNMT3A or negative control. PC: positive control; NC: negative control. (F) MiR‐200b levels were analysed by qRT‐PCR following transfection with si‐DNMT3A or negative control. Data are represented as mean ± SD from three independent experiments. DAC, decitabine. *P < 0.05; **P < 0.01

Epigenetic modulation of the miR‐200 family is associated with EMT and stem‐like cells transition of breast cancer cells.^18^ To confirm DNA methylation is involved in miR‐200b repression in TNBC cells, we first treated MDA‐MB‐231 and BT549 cells with a demethylation drug, DAC. QRT‐PCR analysis showed that miR‐200b expression was elevated following DAC treatment, suggesting that DNA methylation may be responsible for miR‐200b repression (Figure 2A). Then, we knocked down DNMT3A expression and detected the methylation levels of miR‐200b promoter by bisulfite sequencing and MSP (Figure 2B and C). The results showed that DNMT3A knockdown reduced the miR‐200b methylation levels in MDA‐MB‐231 and BT549 cells (Figure 2D and E). Consequently, miR‐200b expression was restored dramatically (Figure 2F). Meanwhile, the expression of miR‐200a and miR‐429, the other members of miR‐200b/a/429 cluster, was also elevated in cells treated with DAC or DNMT3A siRNA (Figure S2A and B). However, DNMT3A knockdown did not significantly increase miR‐200b expression in MCF‐7 cells (Figure S2C). These results suggested that DNMT3A knockdown increased miR‐200b/a/429 expression via promoter demethylation in TNBC cells.

### 3.3 MYC recruits DNMT3A to miR‐200b promoter region inducing gene silencing

**Figure 3:**
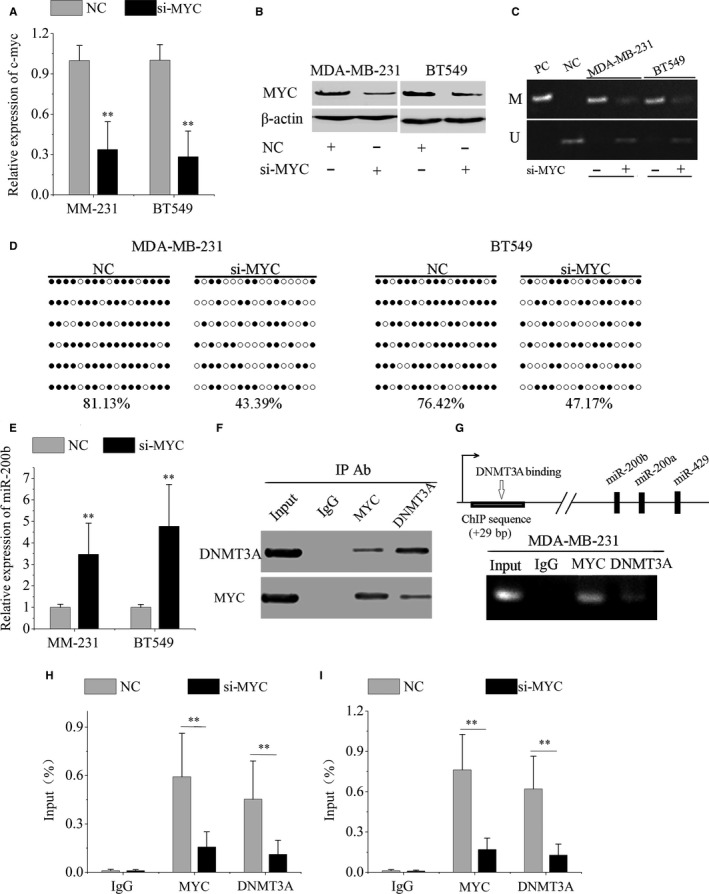
MYC and DNMT3A cooperate in miR‐200b gene silencing. qRT‐PCR (A) and Western blot (B) assay of MYC expression in MDA‐MB‐231 and BT549 cells following transfection with MYC siRNA (si‐MYC) or negative control (NC). (C) The methylation status of miR‐200b promoter region was detected by MSP following MYC knockdown. PC: positive control; NC: negative control. (D) The miR‐200b promoter methylation levels were detected by bisulfite sequencing following MYC knockdown. (E) MiR‐200b levels were increased following MYC knockdown. (F) Co‐immunoprecipitation analysis showed endogenous MYC co‐immunoprecipitated with endogenous DNMT3A in MDA‐MB‐231 cells. (G) A schematic diagram of the DNMT3A binding site in miR‐200b promoter was shown. ChIP assay showed that both MYC and DNMT3A bounded to mir‐200b promoter region in MDA‐MB‐231 cells. Quantitative ChIP assay showed knockdown of MYC led to a decrease in DNMT3A bounding to the promoter of mir‐200b in MDA‐MB‐231 (H) and BT549 cells (I). Data are represented as mean ± SD from three independent experiments. *P < 0.05; **P < 0.01

To investigate whether MYC is involved in epigenetic mir‐200b repression, we knocked down MYC expression with siRNA in MDA‐MB‐231 and BT549 cells (Figure 3A and B). MSP and bisulfite sequencing analysis showed that miR‐200b methylation levels were reduced following MYC knockdown (Figure 3C and D). Subsequently, we found the expression of miR‐200b, miR‐200a and miR‐429 was significantly increased following MYC knockdown (Figure 3E and Figure S3). In addition, DNMT3A knockdown did not significantly reduce MYC levels in TNBC cells (Figure S4). It seemed that DNMT3A knockdown may not increase miR‐200b expression by repressing MYC.

To explore the interaction between MYC and DNMT3A on repressing miR‐200b expression, we performed co‐immunoprecipitation with anti‐MYC antibody and anti‐DNMT3A antibody. The results showed endogenous MYC coimmunoprecipitated with endogenous DNMT3A in MDA‐MB‐231 cells (Figure 3F). Subsequently, we performed ChIP to detect the specific binding of MYC and DNMT3A to mir‐200b promoter region. The results showed that both MYC and DNMT3A were enriched in the mir‐200b promoter region (Figure 3G). Moreover, knockdown of MYC decreased DNMT3A binding to the miR‐200b promoter region (Figure 3H and I). Taken together, MYC may recruit DNMT3A to the miR‐200b promoter region resulting in CpG island hypermethylation and miR‐200b silencing.

### 3.4 MiR‐200b and DNMT3A form a regulatory feedback loop

**Figure 4:**
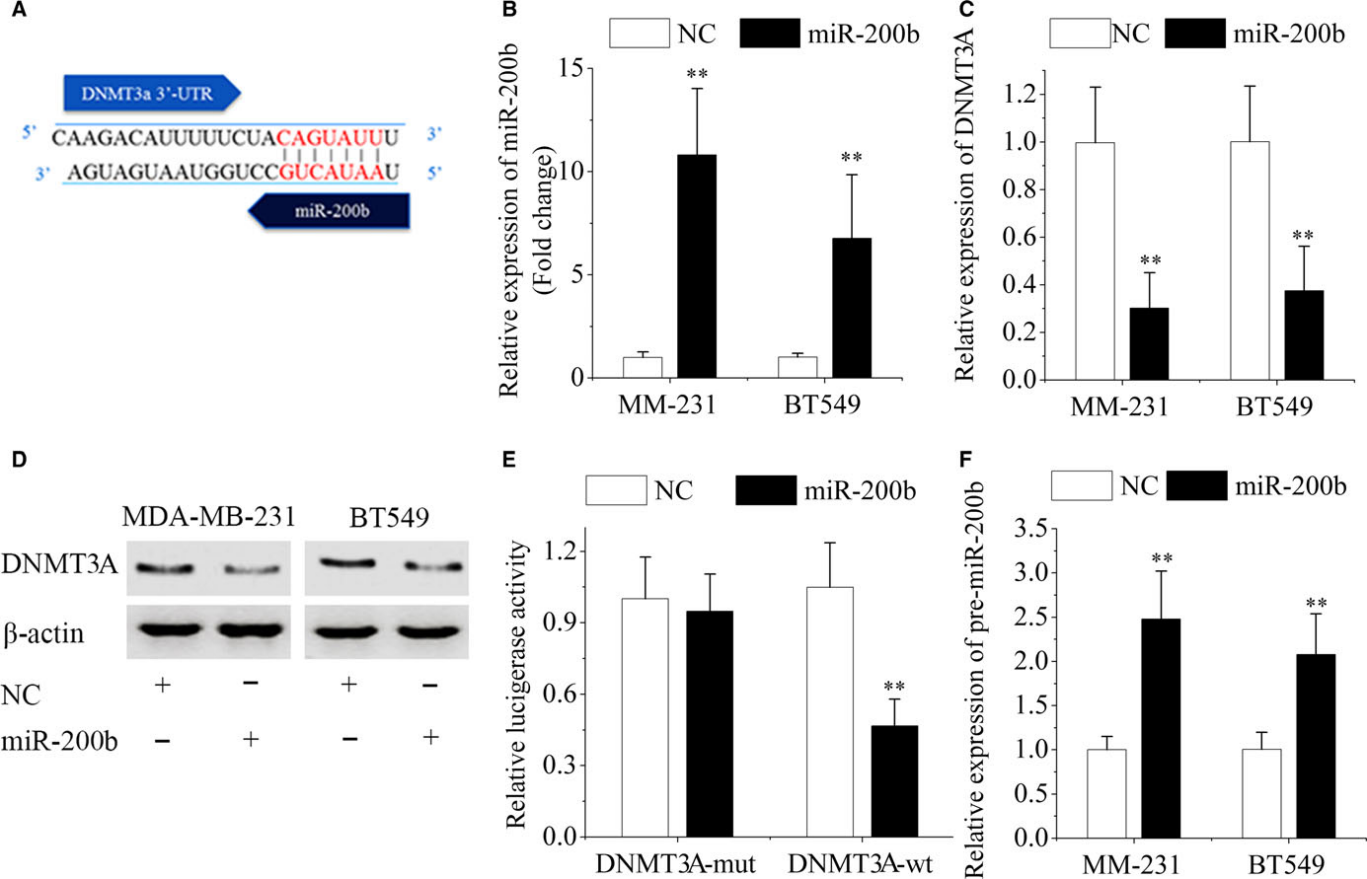
MiR‐200b and DNMT3A form a regulatory feedback loop. (A) The DNMT3A 3′‐UTR sequences possess putative binding site for miR‐200b. (B) MiR‐200b levels in MDA‐MB‐231 and BT549 cells were analysed after transfection with miR‐200b mimics or negative control (NC). (C and D) The expression of DNMT3A was analysed by qRT‐PCR and western blotting. β‐actin was used as internal control. (E) Luciferase reporter assay were performed in MDA‐MB‐231 cells following cotransfection with a luciferase reporter containing DNMT3A‐wt or DNMT3A‐mut together with miR‐200b mimics or negative control. (F) Transfection of miR‐200b mimics increased the pre‐miR‐200b levels in MDA‐MB‐231 and BT549 cells. Data are represented as mean ± SD from three independent experiments. *P < 0.05; **P < 0.01

Using the Targetscan, miRanda and Pictar database, a binding site for miR‐200b was predicted in the 3′‐UTR of DNMT3A mRNA (Figure 4A). To investigate whether miR‐200b inhibits DNMT3A expression, we introduced miR‐200b mimics or negative control mimics into MDA‐MB‐231 and BT549 cells (Figure 4B). The results showed that miR‐200b overexpression reduced the expression of DNMT3A mRNA and protein in TNBC cells (Figure 4C and D). In addition, miR‐200b knockdown increased DNMT3A expression in MCF‐7 cells (Figure S5). Subsequently, we constructed dual‐luciferase reporter vectors with the wild‐type 3′‐UTR of DNMT3A (DNMT3A‐wt) and the mutant 3′‐UTR of DNMT3A (DNMT3A‐mut). The luciferase activity in cells transfected with the DNMT3A‐wt vector was reduced by miR‐200b overexpression, whereas the luciferase activity was not affected with the DNMT3A‐mut vector (Figure 4E). These results indicated that DNMT3A was a direct target of miR‐200b in breast cancer cells. Besides, transfection of miR‐200b mimics dramatically increased the endogenous levels of pre‐miR‐200b (Figure 4F). Above all, there was a regulatory feedback loop between miR‐200b and DNMT3A.

### 3.5 MiR‐200b overexpression or DNMT3A silencing represses EMT and mammosphere formation of TNBC cells

**Figure 5:**
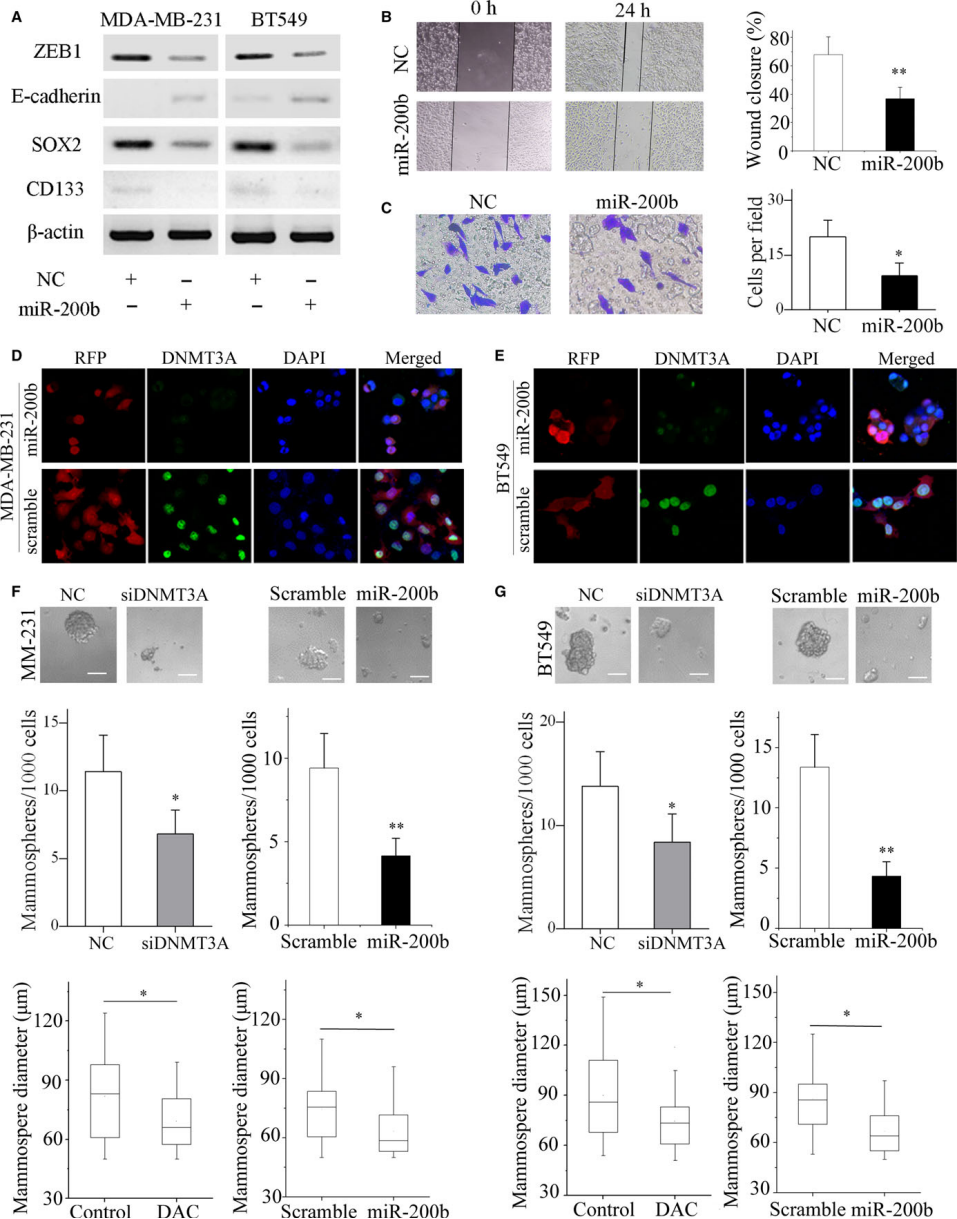
MiR‐200b overexpression represses migration, invasion and mammosphere formation of TNBC cells. (A) ZEB1, E‐cadherin, SOX2, CD133 protein levels in MDA‐MB‐231 and BT549 cells were analysed by western blotting following transfection with miR‐200b mimics or negative control (NC). (B) Wound healing assay showed overexpression of miR‐200b suppressed migration ability of MDA‐MB‐231 cells. Representative images are shown. (C) The invasion ability of MDA‐MB‐231 cells was evaluated by transwell assay. Representative images of the experiments are shown. Original magnification: ×200. The immunofluorescence images of MDA‐MB‐231 (D) and BT549 (E) cells transfected with lentiviral vectors overexpressing miR‐200b or scramble vectors were shown (RFP, pGLVU6/RFP lentiviral vectors). The MDA‐MB‐231 (F) and BT549 (G) cells with DNMT3A knockdown or miR‐200b overexpression formed fewer and smaller mammospheres than the control or scramble. Representative images of the assays are shown (scale bars, 50 μm). *P < 0.05; **P < 0.01

The miR‐200 family plays an important role in EMT and stem‐like properties of breast cancer cells as previously described.^29^, ^30^ First, we investigated the effect of miR‐200b overexpression on a cohort of EMT and stemness regulators and markers. Western blot analysis showed miR‐200b overexpression decreased ZEB1, SOX2, and CD133 expression, and increased E‐cadherin expression (Figure 5A). In addition, miR‐200b overexpression reduced Vimentin and N‐cadherin levels (Figure S6A). ZEB1 and SOX2 were proved to be direct targets of miR‐200 family, while the CD133 3′‐UTR possesses a binding site for miR‐200b according to miRanda database.^16^, ^31^, ^32^ Migration and invasion assays showed that enforced miR‐200b expression markedly inhibited the migration and invasion ability of MDA‐MB‐231 cells (Figure 5B and C). In contrast, miR‐200b knockdown promoted cell migration and invasion (Figure S6B). In addition, DNMT3A silencing inhibited the migration and invasion ability of MDA‐MB‐231 cells (Figure S6C).

To investigate the effect of miR‐200b overexpression on the mammosphere formation and cell growth in vivo, we transfected MDA‐MB‐231 and BT549 cells with miR‐200b‐overexpressing lentivirus vectors (pGLVU6/RFP) or scramble vectors. The immunofluorescence results showed the transfection of the lentivirus vectors into MDA‐MB‐231 and BT549 cells was successful and led to DNMT3A repression (Figure 5D and E and Figure S7A). The number of mammospheres represents the self‐renewal ability of the cells, and the diameter of mammospheres indicates the self‐renewal ability of each sphere‐generating cell.^33^, ^34^ The cells overexpressing miR‐200b formed fewer and smaller mammospheres than the scramble groups. Notably, DNMT3A silencing also resulted in lower mammosphere‐forming efficiency (Figure 5F and G). In addition, mouse xenograft experiments showed that miR‐200b overexpression inhibited tumour growth in vivo (Figure S7B, C and D). Furthermore, western blotting analysis showed that miR‐200b overexpression may inhibited EMT and self‐renewal ability in vivo (Figure S7E). Above all, miR‐200b overexpression or DNMT3A silencing suppressed the EMT and mammosphere formation of TNBC cells.

## 4 DISCUSSION

The miR‐200 family expression levels have been proved to be different among breast cancer subtypes.^27^, ^35^ Using the public prediction database, MiR‐200b/c/429 is predicted to directly targeting DNMT3A. Furthermore, miR‐200b has a stronger inhibitory effect on mammosphere formation than other members of miR‐200 family.^36^ Although all miR‐200 family members were repressed in TNBC cell lines, only miR‐200b repression (~100‐fold) was consistently observed as previously reported.^30^ As a result, miR‐200b was selected to be main research molecular. In this study, miR‐200b expression in TNBC tissues was inhibited compared with other breast cancer subtypes, while DNMT3A and MYC expression were elevated significantly. Our study showed that the TNBC cells showed a lower expression level of miR‐200b than other breast cancer cells and normal breast cells as previously described.^30^ Furthermore, we explored the interaction of MYC, DNMT3A and miR‐200b in TNBC cells, and revealed that miR‐200b was epigenetically suppressed by MYC and DNMT3A‐mediated promoter methylation.

As a transcription factor, MYC can promote some target genes expression by dimerizing with Max, and repress others through interacting with Miz‐1, Sp1 and Smad2.^37^, ^38^, ^39^ Recent studies have shown that MYC is associated with epigenetic regulators in silencing miRNAs. In hepatocellular carcinoma, MYC repressed miR‐101 expression by recruiting EZH2 to the promoter regions of miR‐101.^40^ During Helicobacter pylori‐related carcinogenesis, MYC, DNMT3B and EZH2 interacted with each other and led to let‐7c silencing by inducing histone methylation and DNA hypermethylation.^41^ However, the role of MYC in epigenetically regulating miR‐200 expression is still unclear.

A previous study has demonstrated that MYC represses p21Cip1 transcription through recruitment of DNMT3A.^42^ MYC directly binds to and represses the miR‐200b promoter in endometrial carcinoma cells.^43^ Furthermore, DNMT3A knockdown increases miR‐200b expression.^44^ Then, we wondered whether MYC is responsible for the epigenetic repression of miR‐200b via interacting with DNMT3A. Here, we reported that MYC was associated with DNMT3A in MDA‐MB‐231 cells. The complex of MYC and DNMT3A co‐occupied the miR‐200b promoter and resulted in miR‐200b silencing dependent on DNMT3A‐mediated promoter methylation.

However, there was a discrepancy between BSP results in breast cancer cell lines and TCGA methylation sequencing data in breast cancer tissues. This may be attributed to the different CpG islands included in BSP (Region A) and TCGA methylation sequencing (Region B) (Figure S8). A previous study has demonstrated that the miR‐200b promoter possesses many CpG islands which were methylated differently.^45^ It is unclear whether methylation levels of these CpG sites (Region B) are associated with miR‐200b expression. The incorporation of more CpG sites into methylation sequencing will contribute to the validation of the conclusion.

Moreover, we demonstrated miR‐200b directly inhibited DNMT3A expression and then formed a feedback loop, which contributed to further miR‐200b repression and DNMT3A overexpression by a self‐reinforcing system.^46^ We also try to validate the conclusion in other breast cancer cell lines such as MCF‐7. Because of the low methylation level of miR‐200b promoter, the feedback loop did not exist in MCF‐7 cells. We demonstrated that miR‐200b overexpression or DNMT3A knockdown inhibited EMT and self‐renewal of TNBC cells.^11^ Thus, miR‐200 repression or DNMT3A overexpression may be responsible for the aggressive behaviours of TNBC, paving the way for developing novel targeted agents and designing therapeutic strategy. Delivering chemically modified miRNA mimics by nanoparticles seems to be a promising therapy to overcome the repression of tumour suppressor miRNAs. Another therapy strategy is the drugs that can affect miRNAs regulation.^47^ Interestingly, a latest study has shown that HDAC inhibitors for epigenetic regulation of miR‐200 are potential therapy against TNBC.^48^ However, the instability of delivery in vivo remains a challenge of the miRNA‐based therapy.

To be concluded, we found that the feedback loop between miR‐200b and DNMT3A played an important role in EMT and mammosphere formation of TNBC cells. Importantly, we demonstrated that miR‐200b was epigenetically silenced by MYC and DNMT3A‐mediated DNA methylation. We believed the newly identified feedback loop provides a new insight into the pathogenesis of TNBC, and represents potential therapeutic targets for the treatment of TNBC.

## CONFLICT OF INTEREST

The authors declare no conflicts of interest.
