# Supplementary figures and images for: MYC and DNMT3A‐mediated DNA methylation represses microRNA‐200b in triple negative breast cancer
Source: J Cell Mol Med. 2018 Oct 16;22(12):6262–74. doi: 10.1111/jcmm.13916 (PMC6237581; doi:10.1111/jcmm.13916)

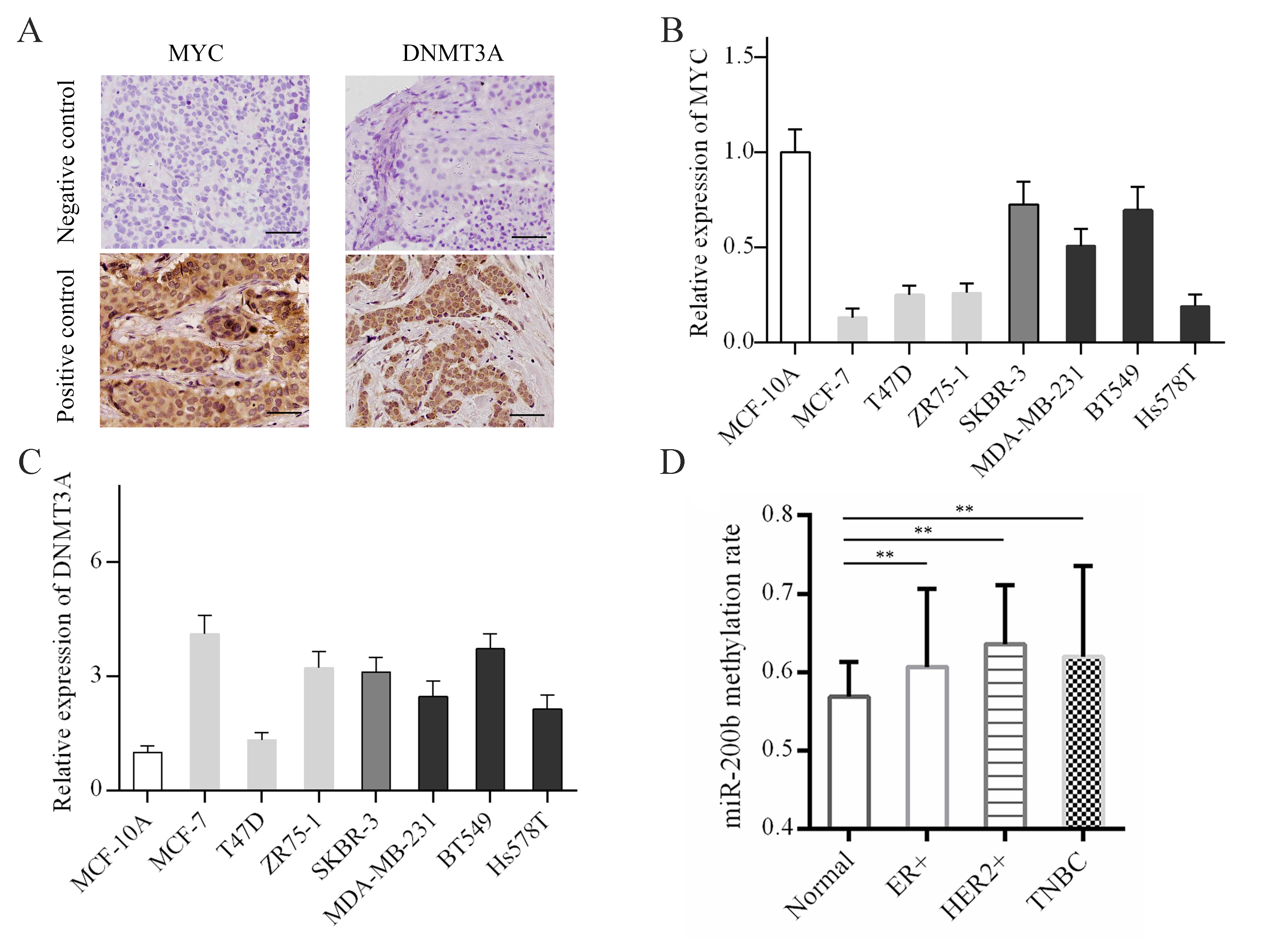

Supplement: Supplementary file 1 [file JCMM-22-6262-s001.tif]

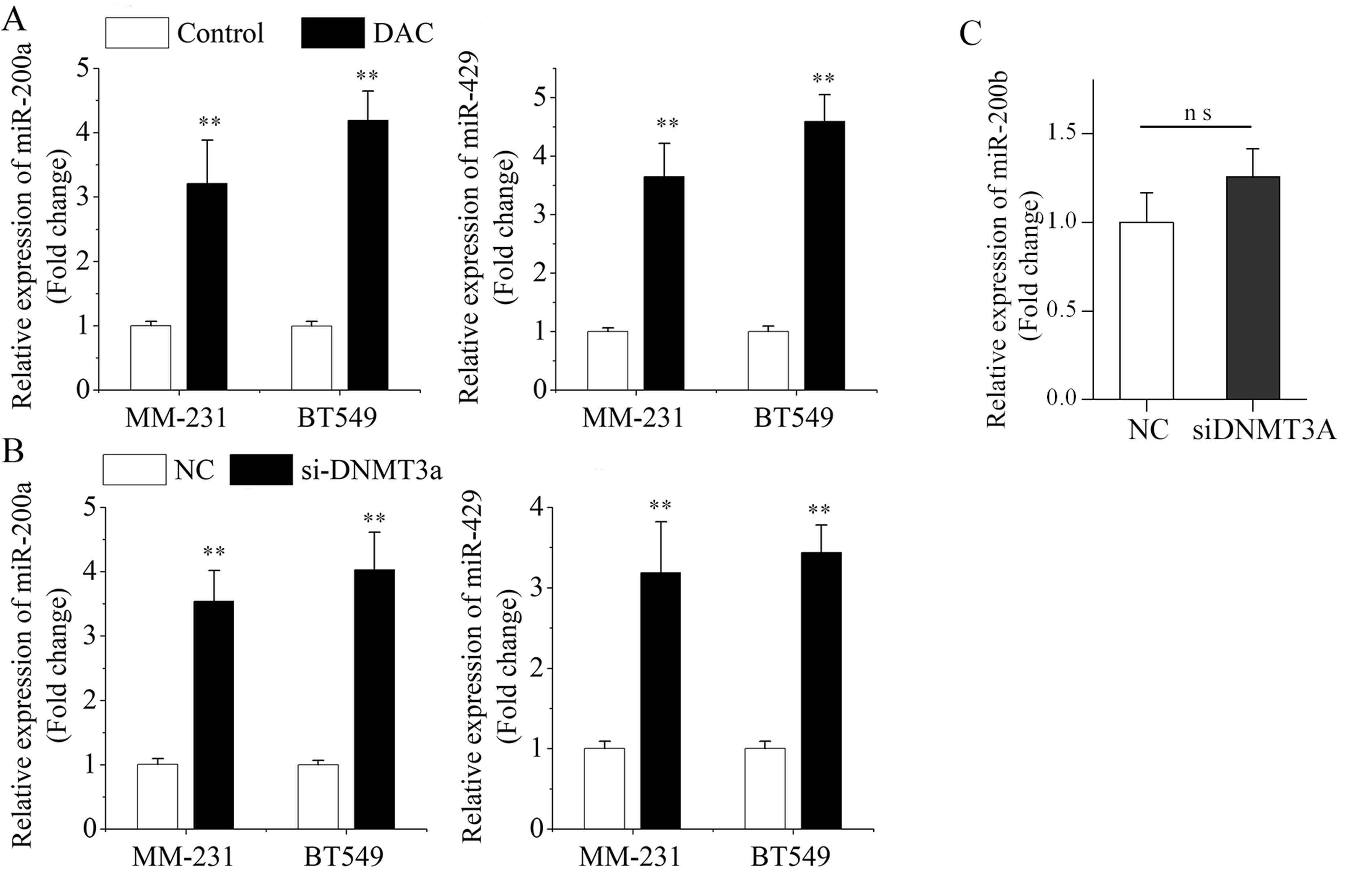

Supplement: Supplementary file 2 [file JCMM-22-6262-s002.tif]

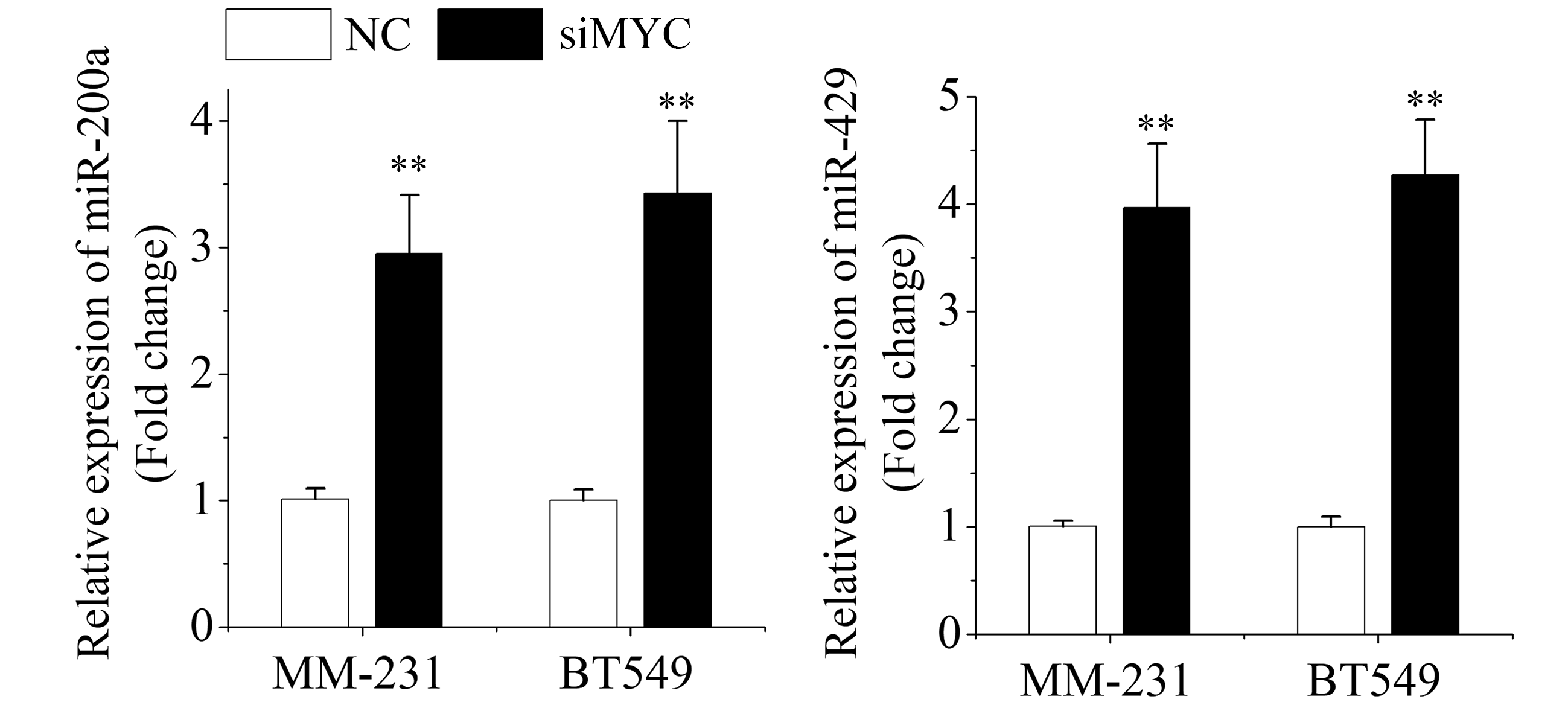

Supplement: Supplementary file 3 [file JCMM-22-6262-s003.tif]

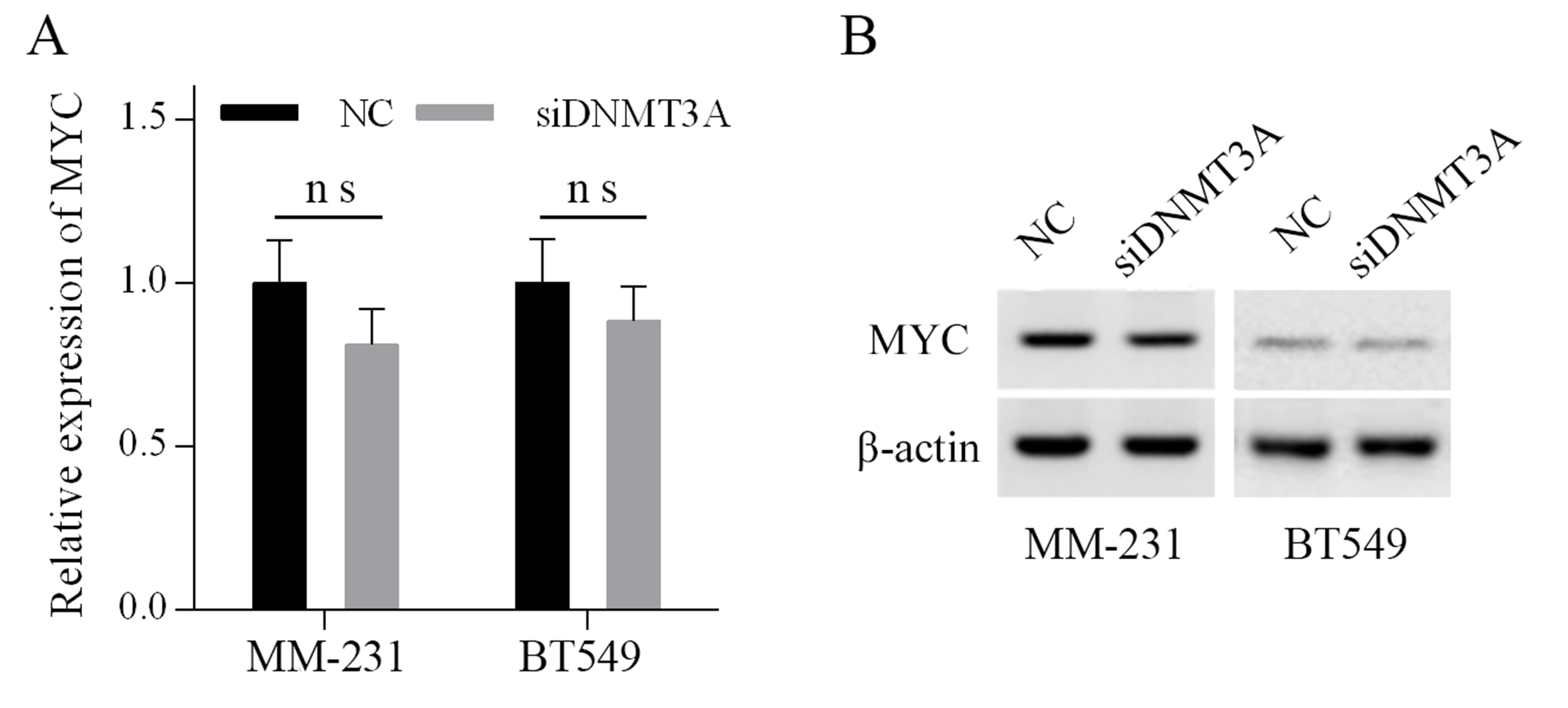

Supplement: Supplementary file 4 [file JCMM-22-6262-s004.tif]

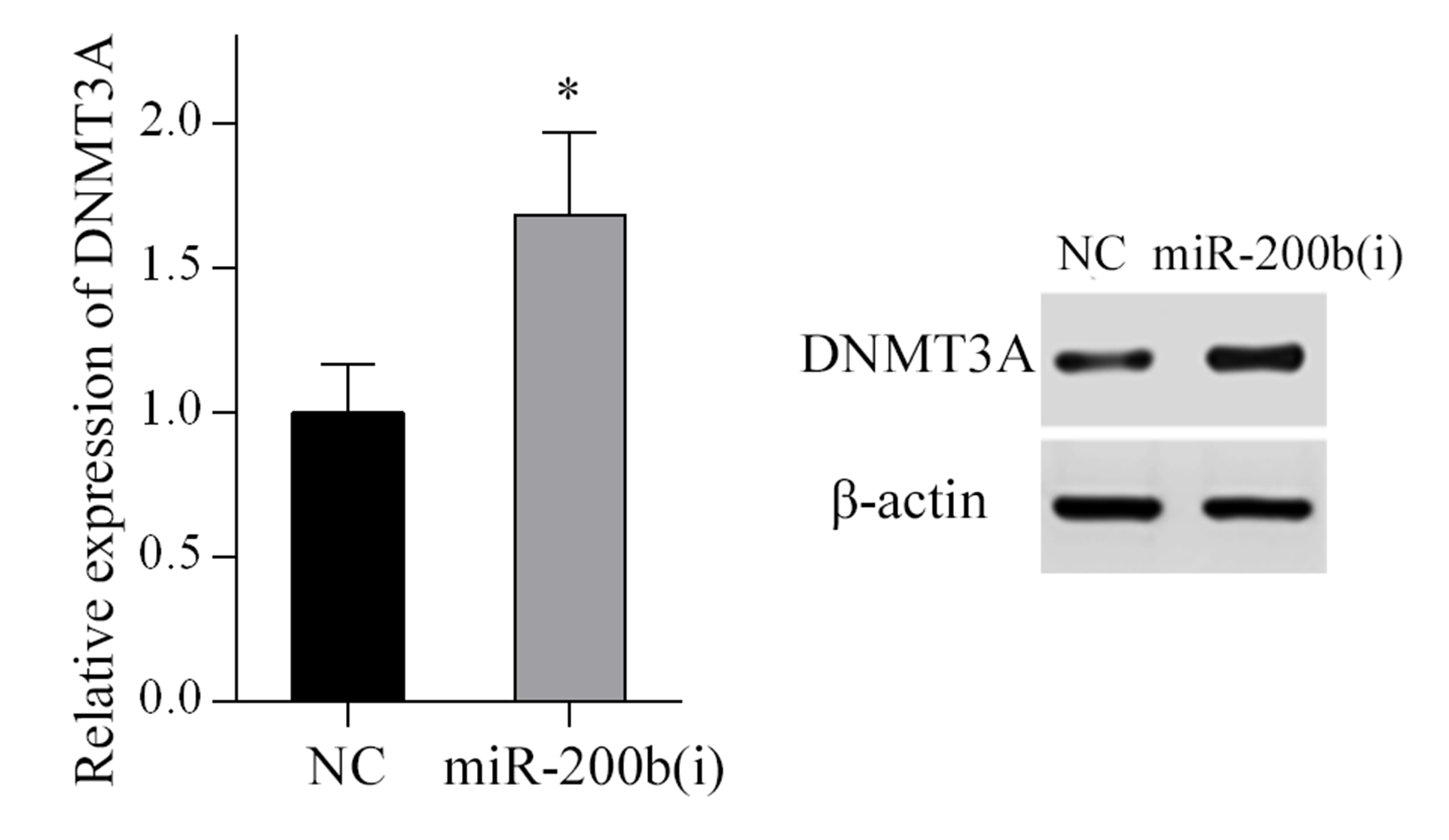

Supplement: Supplementary file 5 [file JCMM-22-6262-s005.tif]

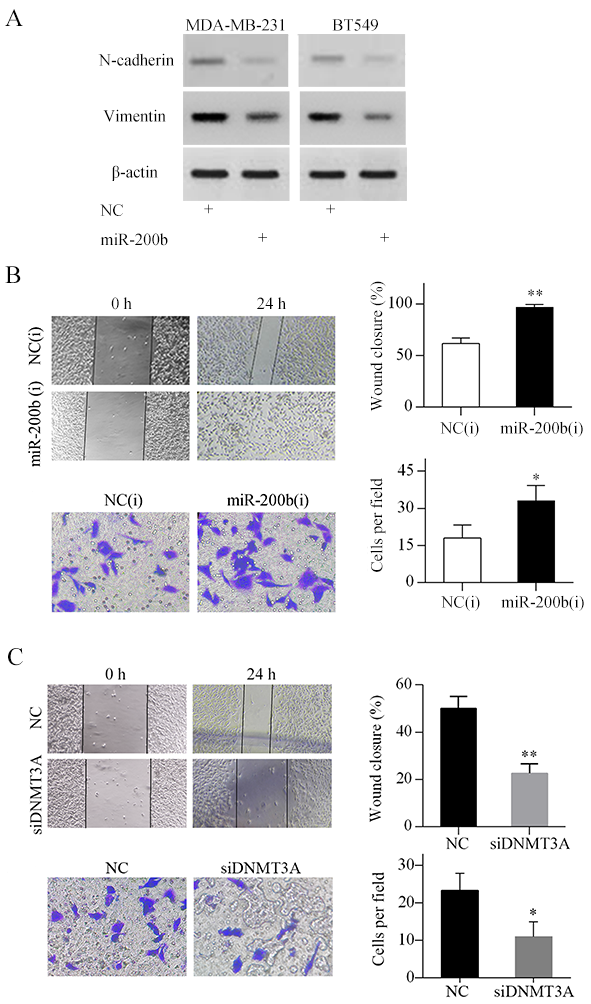

Supplement: Supplementary file 6 [file JCMM-22-6262-s006.tif]

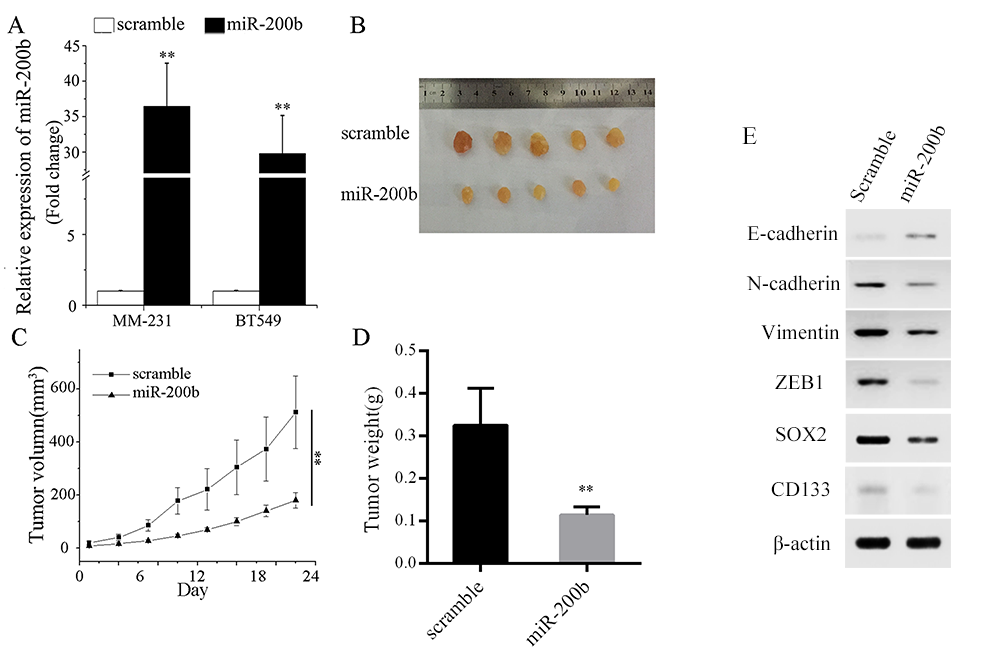

Supplement: Supplementary file 7 [file JCMM-22-6262-s007.tif]

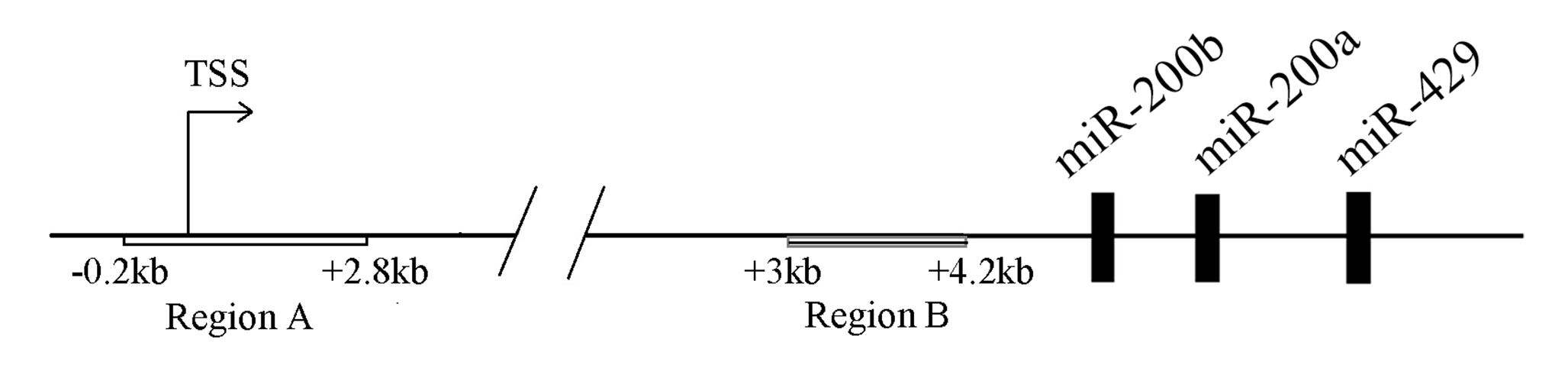

Supplement: Supplementary file 8 [file JCMM-22-6262-s008.tif]
