# Supplementary material for: MYC and DNMT3A‐mediated DNA methylation represses microRNA‐200b in triple negative breast cancer
Source: J Cell Mol Med. 2018 Oct 16;22(12):6262–74. doi: 10.1111/jcmm.13916 (PMC6237581; doi:10.1111/jcmm.13916)
Supplement: Supplementary file 9 [file JCMM-22-6262-s009.docx]

Table 1 Clinicopathologic characteristics of patients

| Characteristics | ER+(n=20) | HER2+(n=13) | TNBC(n=31) |  |
| --- | --- | --- | --- | --- |
| Age(mean±SD) | 50.2±10.8 | 49.3±13.6 | 47.9±10.3 |  |
| Histologic grade |  |  |  |  |
| 1-2 | 10 | 5 | 12 |  |
| 3 | 10 | 8 | 19 |  |
| TNM stage^a^ |  |  |  |  |
| I -II | 13 | 9 | 23 |  |
| III | 7 | 4 | 8 |  |
| Lymph node |  |  |  |  |
| Positive | 15 | 5 | 22 |  |
| Negative | 5 | 8 | 9 |  |
| Histologic type |  |  |  |  |
| Ductal | 17 | 12 | 26 |  |
| Lobular | 3 | 1 | 5 |  |

^a^ All the tumors were proved to be primary breast cancer.

There was no significant difference among the three groups.
