# Supplementary material for: MYC and DNMT3A‐mediated DNA methylation represses microRNA‐200b in triple negative breast cancer
Source: J Cell Mol Med. 2018 Oct 16;22(12):6262–74. doi: 10.1111/jcmm.13916 (PMC6237581; doi:10.1111/jcmm.13916)
Supplement: Supplementary file 10 [file JCMM-22-6262-s010.docx]

Table S2 Primer sequences used in the study

| MYC | Forward primer | TTCGGGTAGTGGAAAACCAG |
| --- | --- | --- |
|  | Reverse primer | CAGCAGCTCGAATTTCTTCC |
| DNMT3A | Forward primer | TAAGCTGGAGCTGCAGGAGT |
|  | Reverse primer | GGAAACCAAATACCCTTTCCA |
| U6 | Forward primer | CTCGCTTCGGCAGCACA |
|  | Reverse primer | AACGCTTCACGAATTTGCGT |
| ACTB | Forward primer | CCTTCTACAATGAGCTGCGT |
|  | Reverse primer | CCTGGATAGCAACGTACATG |
| miR-200b | Forward primer | GCGGCTAATACTGCCTGGTAA |
|  | Reverse primer | GTGCAGGGTCCGAGGT |
| Pre-miR-200b | Forward primer | CCAGCTCGGGCAGCCGTGGC |
|  | Reverse primer | TGACGGCGGAGCCCTGCACG |
| DNMT3A-3’UTR clone | Forward primer | GCTCTAGACGAAAAGGGTTGGACATCAT |
|  | Reverse primer | GCTCTAGAGCCGAGGGAGTCTCCTTTTA |
