# Supplementary material for: MYC and DNMT3A‐mediated DNA methylation represses microRNA‐200b in triple negative breast cancer
Source: J Cell Mol Med. 2018 Oct 16;22(12):6262–74. doi: 10.1111/jcmm.13916 (PMC6237581; doi:10.1111/jcmm.13916)
Supplement: Supplementary file 11 [file JCMM-22-6262-s011.docx]

**Supplementary Methods**

Mouse xenograft model

MDA-MB-231 cells stably transfected with miR-200b-overexpressing or scramble lentivirus were suspended in PBS at a concentration of 1 × 10^7^ cells/ml. Then 200 μl cancer cells suspension was inoculated subcutaneously into the dorsal flanks of nude mice. Each group contained 5 nude mice. The tumour size was measured every three days. Tumors were measured were calculated using the formula: V=1/2×Lengh×Width^2^. After 22 days, the mice were euthanized, and the tumors were weighed. The animal experiments were approved by the Experimental Animal Committee of Xian Jiaotong University.
